# Evaluating iodine deficiency and goiter in hilly areas of District Poonch Azad Jammu Kashmir

**DOI:** 10.12669/pjms.40.7.8784

**Published:** 2024-08

**Authors:** Abdul Khaliq, Mehwish Fayaz, Imran Hayat, Muhammad Abbas

**Affiliations:** 1 Prof. Dr. Abdul Khaliq, Professor Department of Soil and Environmental Sciences, Faculty of Agriculture, University of Poonch Rawalakot, Rawalakot Poonch, Azad Jammu Kashmir, Pakistan; 2 Mehwish Fayaz, Lecturer, Department of Eastern Medicine and Surgery, University of Poonch Rawalakot, Rawalakot Poonch, Azad Jammu Kashmir, Pakistan; 3 Dr. Imran Hayat, Professor Department of Food Sciences and Technology, University of Poonch Rawalakot, Rawalakot Poonch, Azad Jammu Kashmir, Pakistan; 4 Muhammad Abbas, Department of Soil and Environmental Sciences, Faculty of Agriculture, University of Poonch Rawalakot, Rawalakot Poonch, Azad Jammu Kashmir, Pakistan

**Keywords:** Iodine Deficiency, Goiter, Urine iodine concentrations

## Abstract

**Background & Objectives:**

Iodine deficiency is considered as a global challenge, even after decades of efforts to solve the issue. Our objective was to assess the iodine deficiency status and associated prevalence of goiter in population groups (school-age children and women) from rural areas of District Poonch, and to assess the determinants of iodine deficiency in the area.

**Methods:**

Cross-sectional study was conducted in District Poonch Azad Jammu and Kashmir from 25 April 2022 to 30 June 2023. A total of 150 goiter patients from different villages of the District Poonch was included. Their urinary iodine concentration and goiter prevalence was assessed. Using palpation techniques, a trained and experienced public health officer assessed the presence of goiters based on WHO/ UNICEF/ICCIDD criteria. Descriptive statistics were computed for continuous variables and presented in frequency and percentage, based on the distributional characteristics of the data. chi-square was used to check association between socio-demographic factors and goiter. It was a HEC Project No.: 20-16988/NRPU/R&D/HEC/2021.

**Results:**

Iodine status and associated goiter prevalence was high and 59.3% of them were severely iodine deficient. Within the district, the highest severe iodine deficiency was observed in 81.1% goiter patients of the Rawalakot subdivision, Hajira and Abbaspur subdivisions. Regarding goiter status 40% of the patients were classified with palpable-visible goiter and 56% were characterized with visible but nodular goiter.

**Conclusion:**

Study showed that there was a severe iodine deficiency and associated goiter prevalence in the area. Policymakers should take actions for future to overcome iodine deficiency in future.

## INTRODUCTION

Despite decades of significant attempts to address the issue, iodine deficiency remains a severe concern across the world. In 2008, economists participating in the Copenhagen Consensus rated universal salt iodization (USI) third among the top ten obstacles to enhancing global wellbeing. The International Child Development Steering Group has identified iodine deficiency as one of four critical global health concerns associated with delayed child development.^1^ About, 241 million children worldwide more than 1.88 billion people do not consume enough iodine. These children are vulnerable to the negative consequences of iodine deficiency, which include endemic goiter, cretinism, intellectual impairment, growth retardation, neonatal hypothyroidism, miscarriage, and infant mortality. Iodine deficiency diseases (IDDs) continue to pose a problem to nations that are both developed and developing.^2^ Iodine deficiency has been historically public health issues in the nations of the Eastern Mediterranean Region (EMR), namely in Afghanistan, Algeria, Iraq, Iran, Morocco, Saudi Arabia, Sudan, Yemen, and Pakistan. Europe having the greatest percentage of children worldwide (44%) with insufficient iodine supplies.^3^

In the absence of adequate iodine, the levels of thyroid stimulating hormones (TSH) remain elevated, leading to enlargement of the thyroid gland (goiter) that reflects the body’s attempt to trap more iodine from the circulation and produce thyroid hormones. Iodine deficiency diseases (IDDs) impact around 30% of the global population. Goiter is usually the earliest clinical sign of iodine deficiency claimed that 70% population of the mountainous areas of upper Punjab, Khyber Pakhtunkhwa, Naran, Kathan, Balakot, Hazara, Waziristan and Azad Jammu and Kashmir are facing the problem of severe iodine deficiency.^4^

About half of the world’s population suffers from micronutrient malnutrition, especially iron and iodine, which is mainly associated with low dietary intake of micronutrients with less diversity of food. Pakistan is considered to be severely iodine deficient country with its 70% population at risk of iodine deficiency, especially in mountainous areas.^5^ The country-based survey reports on iodine status had shown that 70% population of the mountainous areas of upper Punjab, Khyber Pakhtunkhwa, Naran, Kaghan, Balakot, Hazara, Waziristan and Azad Jammu and Kashmir are facing the problem of severe iodine deficiency.^6^ Consequently, 2.1 million children are still born each year with mental disorders in Pakistan due to iodine deficiency in pregnant women (APP 2013; ICCIDD 2013). Pakistan National Nutritional Survey (conducted by Agha Khan University, Karachi in 2011) reported the highest percentage of children in Gilgit and Azad Jammu& Kashmir was found severely iodine deficient. The origins of this widespread iodine deficiency in the populations of Pakistan are dietary. Cereal grains (e.g. wheat) are poor sources for many micronutrients including iodine. In a study conducted in 2015, it was reported a daily iodine intake of 25.4 µg from wheat grains which is far below than that recommended by World Health Organization (WHO; i.e.150 µg day^-1^) for adults to prevent IDDs.^7^ This nominal intake of iodine is alarming since 60 % of Pakistani households directly rely on wheat grains and its products.

Pregnant and lactating women are considered as the most vulnerable groups for iodine deficiency because of their elevated iodine requirements. Around 60% pregnant women in Europe have inadequate iodine intake. Similarly, more than 50% of women of child-bearing age were found iodine deficient in United Kingdom. Pregnant/ lactating women and children living in low-to middle income countries, are most vulnerable age groups.^8^ The WHO (2007) recommended daily iodine intakes of 250 µg for pregnant and lactating women, from both supplements and dietary sources.

Iodine status is also associated with the eating habits and socioeconomic status of the rural communities. In developing countries, the diet mainly consists of staple foods that are of low nutritional values and do not meet the daily dietary requirements of these essential micronutrients. The iodine deficiency is inversely related to an increase in average monthly income of households and their educational level.^9^ The severity of iodine deficiency increases the occurrence of poor pregnancy outcomes such as miscarriage, stillbirth and increased rate of infant mortality. The fetal brain development is sensitive even to a minor adjustment in thyroid hormone.

Till to date, information about current nutritional status of iodine and associated prevalence of goiter in young children and pregnant women of the region is missing. Therefore, this project is planned to assess the prevalence of iodine deficiency disorders (IDDs) and identify endemic goiter disease in population groups through systematic survey of people, soil, water and crops to provide a baseline data for future monitoring and recommending site specific preventive measures that could help to reduce public health problem.

## METHODS

A descriptive cross-sectional was conducted in District Poonch Azad Jammu and Kashmir from 25 April 2022 to 30 June 2023.

**Fig.1:**
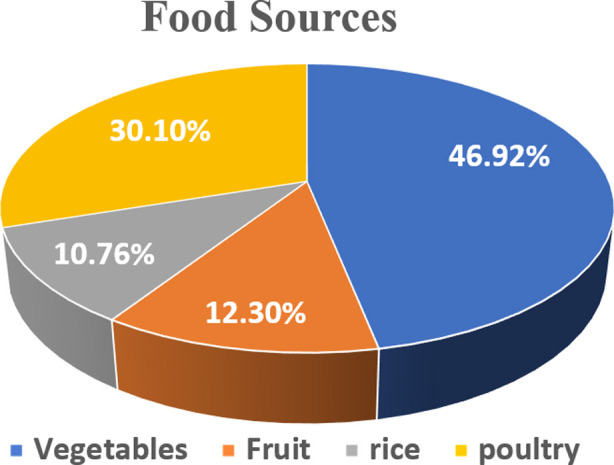
Proportion of dietary food sources utilized by goiter patients.

The detailed survey of more than Hundred (1000 villages of District Poonch, Azad Jammu and Kashmir (Fig.1) was carried out to assess the iodine deficiency status and associated goiter prevalence in population groups of the rural area. The target was established to select a total of 150 goiter patients from different villages of the District Poonch. Fifty patients preferably women and school age children from each of the three subdivisions (Abbaspur, Hajira and Rawalakot) of the District Poonch, with thyroid swelling were contacted and enrolled for this study. The information was collected from the selected patients by interviewing them personally and by involving health workers from health units and hospitals of the area, after obtaining written consent of each patient.

### Ethical Approval

The study was approved by the Ethics Committee (Ref No. UPR/05/59/2021; dated Sep. 5, 2021).

Data was collected using a questionnaire designed to inquire about patients’ general socio-demographic data, including goiter physical appearance, age, occupation, gender, residential area, economic status, educational, and clinical manifestations, among other considerations, were considered.^10^ The food intake data of households were recorded to assess daily dietary iodine intake of populations in the study area. The food items were aggregated/ categorized into 6 sub-groups such as milk and milk products (Yogurt, cheese, curd, butter), meat (beef, mutton and poultry birds), fruit (local and marketed), vegetables (leafy, tuberous, others), cereals (wheat, rice, maize) and salad. The detailed information related to the age, gender, major symptoms (Weight gain, fatigue, irritability etc.), psychological status and physical examination of the patients was also collected during the survey. To accomplish this, the questionnaire comprising of all the required information’s was designed, printed out and carefully filled during the survey. Using palpation techniques, a trained and experienced public health officer assessed the presence of goiters based on WHO/ UNICEF/ICCIDD criteria.^11^ Therefore, Grade 0: stand for not visible, not palpable;Grade: I stand for palpable, not visible; and Grade: II stands for visible and palpable.

### Inclusion & Exclusion Criteria

Inclusion criteria was pregnant women and school going children, who were willing to participate. In the study, patients with serious physical and mental illnesses were excluded as it was difficult to obtain data and measurements from them. The statistical analysis was conducted using the SPSS software version 17.0. Descriptive statistics were computed for continuous variables and presented in frequency and percentage, based on the distributional characteristics of the data.

### Statistical analysis

It was done through chi-square to check association between socio-demographic factors and goiter. The study was approved by HEC the Project No.: 20-16988/NRPU/R&D/HEC/2021.

## RESULTS

### Income Status and Dietary Habits of Enrolled Participants

The survey revealed that the average monthly income of households/ families in the study area varied between PKR. 20,000-150,000. The families earning the highest income had access to the expensive food items including milk, milk products and meat compared with the lowest income earning households, who obtained most of their calories from locally cultivated staple crops such as wheat, maize and rice. Only 5-10% peoples had good quality nutrition and the remaining 90-95% are resource-limited families with low dietary intakes. This indicated that quality wise, the diet of majority of affected populations is nutrient-deficient. These results revealed that improvement in income is the most important factor in rural areas for evaluating population’s access to the key nutrients especially for which the median person is most deficient. The survey report indicated that the fruits and vegetables access accounted for 12.3 and 46.9%, respectively, of the total dietary food consumption by the affected participants (Fig.1). Similarly, 30% participants (39/130) in the study area had a direct access to the poultry meat. A significant proportion of the respondents indicated that the cereals are the major source of their daily dietary intake.

The estimates had shown that only a small proportion (10.76%) of the enrolled participants was consuming rice and rest of the majority (85-90%) was relying on locally available wheat or maize food sources. Overall, knowledge regarding health implications of iodine deficiency was very poor especially in women having low literacy. Around 60% enrolled women of Rawalakot, 81% of Hajira and 92% of the Abbaspur sub-divisions were not aware about the health problems associated with low iodine intake. Apparently, the demographic characteristics of the study area, socioeconomic status of populations, low quality of drinking water, very limited access to nutrient rich diet and eating habits of the families appear to be the main causes of iodine deficiency in the study area.

### Assessment of Goiter Prevalence

The results (Tables I & II) indicated that all of the 130 participants (male and female) of different age groups enrolled in our study were identified as goiter patients. As far as their goiter status is concerned, 40% (52/130) of the patients were classified with palpable-visible goiter and 56% (73/130) were characterized with visible but nodular goiter. Only 4% (5/130) of the total patients were fell under the category of goiter having all the three (palpable, visible and nodular) characteristics of goiter. In our study, we found goiter patients even of high income but they are utilizing water of local springs. The lower concentrations of Iodine-3 (IO^-^_3_) in the drinking water might be the possibility of goiter development. Thyroid nodules (TNs) appear more prevalent in iodine-deficient regions where high proportion of Iodine-3 (IO^-^_3_) frequently occurs in groundwater.

**Table-I:** Iodine levels of goiter patients identified from District Poonch of Azad Jammu and Kashmir (based on Median UIC, n=130).

Subdivision	Total reported Patients (No’s)	Severe iodine deficiency (median UI; < 25 µg L^-1^)	Moderate iodine deficiency (median UI; 25-60 µg L^-1^)	Mild iodine deficiency (median UI; 60-120 µg L^-1^)	Optimal iodine intake (median UI; 120-220 µg L^-1^)	Mild iodine excess (median UI; 220-400 µg L^-1^)
Rawalakot	37	30 (81.1%)	07 (18.9%)	0	0	0
Hajira	25	15 (60.0%)	09 (36.0%)	0	0	01(4.0%)
Abbaspur	68	32 (47.1%)	34 (50.0%)	02 (2.9%)	0	0

Total	130	77 (59.3%)	50 (38.5%)	02 (1.5%)	0	01 (0.77%)

**Table-II:** Association of socio-demographic factors with iodine deficiency.

Variable	X2	Df	P-value
Gender	17.19	4	0.012
Residential Area	14.77	3	0.004
Education	17.01	3	0.003
Age	15.22	4	0.040
Iodized salt	18.32	3	0.001
Economic status	13.71	2	0.010

**Fig.2:**
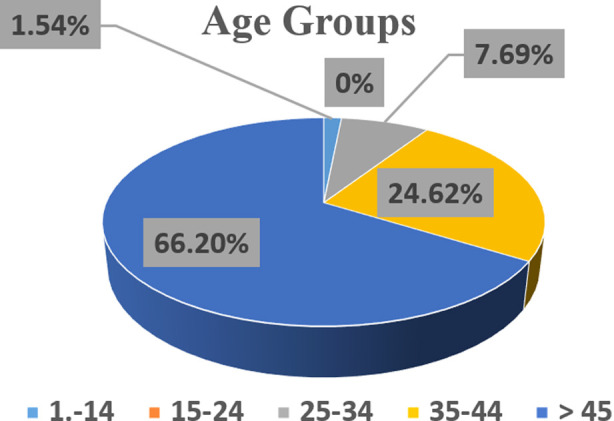
Proportion of goiter in patients of different age groups.

The data presented in Fig.2 indicated the elderly participants belonging to the age group of 45 years or greater were most vulnerable to iodine deficiency, representing 66.2% (86/130) goiter prevalence among all the age groups. The participants falling in age group between 35-44 years were ranked second in order (32/130) by contributing 24.62% to the goiter incidence. The adult participants (between 25-34 years age group) were slightly (7.69%) affected by goiter. No case of goiter was observed and reported in younger participants of age between 15-24 years. In case of school age children (between 5-14 year’s ages) only two goiter cases were noted among all the enrolled participants, indicating a very minute percentage (1.54 %) of the total identified patients. The clinical examination of the patients showed that the patients of all age groups had their goiters of grades one and two, with the exception of one case of age group between 5-14 years.

### Urine Iodine concentrations (UIC) of the Goiter Patients

The estimated median UIC among all goiter patients of different age groups varied between <20 to 301 µg L^-1^. The data regarding the urinary iodine concentrations of the goiter patients indicated that overall, 130 goiter patients were accessed from three subdivisions of the Poonch district during the survey to evaluate their iodine status and associated goiter prevalence and 59.3% (77 out of 130) of them were severely iodine deficient. Within the district, the highest severe iodine deficiency was observed in 81.1% goiter patients of the Rawalakot subdivision followed by Hajira and Abbaspur subdivisions, where 60 and 47% patients, respectively were found severely iodine deficient. Further, the median UIC of the patients showed that 50, 36 and 19% patients from Abbaspur, Hajira and Rawalakot subdivisions are facing the problem of moderate iodine deficiency . The proportion of the mild iodine deficient goiter patients with UIC concentrations between 60-120 µg/L was 1.5% (two out of 130 patients).

The only one lady patient from Mandhole-Tahi village of Hajira subdivision was reported with a median UIC of 301 µg L^-1^, just above the cutoff for mild iodine excess (220-400 µg L^-1^). The patient’s dietary history showed that she is regularly using fish in her diet for the last more than 10-15 years and it might be possible reason of iodine excess and she is at the risk of hyperthyroidism development.

### Association of socio-demographic factors with iodine deficiency

Chi-square was applied to check association between socio-demographic factors and iodine deficiency. There was strong association between gender with the p-value of 0.012, female was more iodine deficient than male. There was strong association between residential area and iodine deficiency with the p-value of 0.004, rural area people were having more iodine deficiency than urban people. Use of Iodized salt was strongly associated with the p-value of 0.001, people using iodized salt were having low iodine deficiency than other. Education and economic status were also strongly associated with the p-value of 0.003 and 0.010 respectively.

## DISCUSSION

Within the district, the highest severe iodine deficiency was observed in 81.1% goiter patients of the Rawalakot subdivision followed by Hajira and Abbaspur subdivisions, where 60 and 47% patients, respectively were found severely iodine deficient. Further, the median UIC of the patients showed that 50, 36 and 19% patients from Abbaspur, Hajira and Rawalakot subdivisions were facing the problem of moderate iodine deficiency it was same as a previous study.^12^ According to a study conducted in India,^13^ the proportion of the mild iodine deficient goiter patients with UIC concentrations between 60-120 µg/L was 2% while our study results indicate that UIC concentration between 60-120 µg/L 1.5% (2 out of 130 patients).

The only female patient at risk was using fish for many years while it might be possible reason of iodine excess and she is at the risk of hyperthyroidism development. Similar results were found in a previous study.^14^ The case of the reported patient is also supported by the findings of previous studies, where they mentioned that the problem of hyperthyroidism develops after more than 10 years of continuous excessive use of iodine rich sources.^15^,^16^ According to previous research the iodine-induced hyperthyroidism (IIH) is the most common complication in almost all iodine supplements in their early phases and long term use of high iodine containing foods.^17^

In our study, the proportion of severely iodine deficient patients (47-81%) is comparable with that of 79.5% earlier report.^18^ Furthermore, other researchers have also documented a prevalence of iodine deficiency ranging from 54.7% to 92.7% based on UI measurements.^19^,^20^

### Strength of the study

It was a laboratory-base design for urinary iodine detection.

### Limitations of the study

There were some limitations of study, as it was a cross-sectional design there was the chances of having non-response recall bias. Despite these limitations, the research highlights the severity of iodine deficiency (laboratory-base) in the region and underscores the need for further investigation and targeted interventions to address this public health issue effectively.

## CONCLUSION

Iodine deficiency (IDD) is a serious community health issue, and lessons can be learned from Pakistan and other countries. All stakeholders should engage in coordinated and regionally accepted initiatives to eradicate IDD. Policymakers should take actions to protect future generations and educate concerned groups about the need of conducting thorough evaluations and estimations of iodine nutritional status. To eradicate IDD, all stakeholders must work together to create to launch consistent awareness campaigns like promotion of iodine supplementation, legislative commitments, raise awareness through various means, effectively monitor salt iodization, implement alternative fortification strategies, emphasizing iodine-rich foods, healthcare infrastructure strengthening, and continuous research and monitoring.

### Authors’ Contribution:

**AK** conceived and designed the project and editing of manuscript.

**MF** did statistical analysis and manuscript writing.

**IH and MA** Data collection and drafting of article or revising it critically for important intellectual content.

**MF** takes the responsibility and is accountable for all aspects of the work in ensuring that questions related to the accuracy and integrity of any part of the work are appropriately investigated and resolved.

## References

[1] Khattak RM, Khattak MN, Ittermann T, Volzke H (2017). Factors affecting sustainable iodine deficiency elimination in Pakistan:A global perspective. J Epidemiol.

[2] Choi J, Oh Y, Chae S, Hong S (2019). Membrane capacitive deionization-reverse electrodialysis hybrid system for improving energy efficiency of reverse osmosis seawater desalination. Desalination.

[3] Faridullah F, Shabbir H, Iqbal A, Bacha AUR, Arifeen A, Bhatti ZA (2022). Iodine supplementation through its biofortification in Brassica species depending on the type of soil. Environ Sci Pollut Res.

[4] Delshad H, Amouzegar A, Mirmiran P, Mehran L, Azizi F (2012). Eighteen Years of Continuously Sustained Elimination of Iodine Deficiency in the Islamic Republic of Iran:The Vitality of Periodic Monitoring. Thyroid.

[5] Ahmad S, Bailey EH, Arshad M, Ahmed S, Watts MJ, Stewart AG, Young SD (2021). Environmental and human iodine and selenium status:lessons from Gilgit-Baltistan, North-East Pakistan. Environ Geochemist Health.

[6] Faridullah F, Shabbir H, Iqbal A, Bacha AUR, Arifeen A, Bhatti ZA (2022). Iodine supplementation through its biofortification in Brassica species depending on the type of soil. Environ Sci Pollut Res.

[7] Zia MH, Watts MJ, Gardner A, Chenery SR (2014). Iodine status of soils, grain crops, and irrigation waters in Pakistan. Environ Earth Sci.

[8] Black RE, Allen LH, Bhutta ZA, Caulfield LE, de Onis M, Ezzati M (2008). Maternal and child undernutrition:global and regional exposures and health consequences. Lancet.

[9] Han MR, Ju DL, Park YJ, Paik HY, Song Y (2015). An Iodine Database for Common Korean Foods and the Association between Iodine Intake and Thyroid Disease in Korean Adults. Int J Thyroidol.

[10] Cocks H, Boelaert K (2013). Assessment of Goiter. Tips and Tricks in Endocrine Surgery.

[11] Masoodi SR, Ali A, Wani AI, Bashir MI, Bhat JA, Mudassar S (2013). Goitre and urinary iodine excretion survey in schoolchildren of Kashmir Valley. Clin Endocrinol.

[12] Chakrabarty A, Pandav CS, Karmarkar MG, Tandon N (2009). Nutritional, Endocrine and Pathological Aspects of Iodine Deficiency Disorders in India. Comprehensive Handbook Iodine.

[13] Kapil U (2015). Iodine nutritional status among adolescent girls in Uttarakhand State, India. Endocr Abstracts.

[14] Andersson M, Karumbunathan V, Zimmermann MB (2012). Global Iodine Status in 2011 and Trends over the Past Decade. J Nutr.

[15] Yadav K, Lohiya A, Kant S, Kumar R, Pandav C (2015). Prevalence of iodine deficiency among adult population residing in Rural Ballabgarh, district Faridabad, Haryana. Indian J Public Health.

[16] Srivastava R, Yadav K, Upadhyay RP, Silan V, Sinha S, Pandav CS (2013). Iodized salt at households and retail shops in a rural community of Northern India. South East Asia J Public Health.

[17] Stanbury JB, Ermans AE, Bourdoux P, Todd C, Oken E, Tonglet R (1998). Iodine-Induced Hyperthyroidism:Occurrence and Epidemiology. Thyroid.

[18] Elahi S, Syed Z, Nagra SA (2004). Status of iodine-deficiency disorders as estimated by neonatal cord serum thyrotropin in Lahore, Pakistan. Nutr Res.

[19] Saira S (2014). Prevalence of Goiter and Iodine Status in 6-12 Years School Children and Pregnant Women of District Charsada, Pakistan. Acta Endocrinologica (Bucharest).

[20] Jahangir M (2015). Prevalence of Goiter and Iodine Nutritional Status in School Age Children of District Karak, Khyber Pakhtunkhwa, Pakistan. Acta Endocrinologica (Bucharest).

